# The history of the future of the Bayesian brain

**DOI:** 10.1016/j.neuroimage.2011.10.004

**Published:** 2012-08-15

**Authors:** Karl Friston

**Affiliations:** The Wellcome Trust Centre for Neuroimaging, UCL, 12 Queen Square, London WC1N 3BG, UK

**Keywords:** Bayesian brain, Functional integration, Effective connectivity, Optimization, Generative models, Predictive coding, Inference, Free energy

## Abstract

The slight perversion of the original title of this piece (The Future of the Bayesian Brain) reflects my attempt to write prospectively about ‘Science and Stories’ over the past 20 years. I will meet this challenge by dealing with the future and then turning to its history. The future of the Bayesian brain (in neuroimaging) is clear: it is the application of dynamic causal modeling to understand how the brain conforms to the free energy principle. In this context, the Bayesian brain is a corollary of the free energy principle, which says that any self organizing system (like a brain or neuroimaging community) must maximize the evidence for its own existence, which means it must minimize its free energy using a model of its world. Dynamic causal modeling involves finding models of the brain that have the greatest evidence or the lowest free energy. In short, the future of imaging neuroscience is to refine models of the brain to minimize free energy, where the brain refines models of the world to minimize free energy. This endeavor itself minimizes free energy because our community is itself a self organizing system. I cannot imagine an alternative future that has the same beautiful self consistency as mine. Having dispensed with the future, we can now focus on the past, which is much more interesting:

## Introduction

A history of scientific ideas is a history of people. What follows is a series of anecdotes about the people who have shaped our current thinking about the brain and, in particular the Bayesian brain. This is necessarily a personal account and some of the anecdotes may or may not be true but they are at least colorful. It should be noted that this account is just one of many perspectives on the Bayesian brain. Furthermore, I have tried to give this account an autobiographical narrative, which means the stories (and their characters) are somewhat selective.

### Prologue

For me, the story starts when I was 8 years old. My mother had sent me outside to play in one of those hot summers of the 1960s. In a Gerald Durrell moment I overturned an old log and found myself absorbed in the antics of some woodlice (miniature armadillo-like bugs) that were fleeing for cover of darkness. After a few minutes observing them closely I had my first (and possibly last) scientific insight: they were not purposefully (mindfully) seeking darkness, they were simply moving faster (in any random direction) when warmed by the sun. Over the next 40 years, I was to draw on this early notion when learning about natural selection, information theory, machine learning and statistical thermodynamics. What follows is a story about some of the people in these disciplines who gave us the Bayesian brain.

### The Bayesian brain

To understand the role of the Bayesian brain in imaging neuroscience, it is necessary to unpack the ideas that it entails. In essence, the Bayesian brain says that we are trying to infer the causes of our sensations based on a generative model of the world. This idea has a long history dating back to the students of Plato and probably most clearly articulated by von Helmholtz (1866). The idea has gathered momentum over the past century, as reflected in the notion of perception as hypothesis testing (Gregory, 1968, 1980; Kersten et al., 2004) and the central role of Bayesian probability theory in computational neuroscience (Dayan et al., 1995; Lee and Mumford, 2003). Particular instances of the Bayesian brain, such as predictive coding (Rao and Ballard, 1998) and optimal decision (game) theory in neuroeconomics have now come to dominate much of neuroscience at the systems level (Knill and Pouget, 2004), particularly in functional imaging. It is important to appreciate that the Bayesian brain hypothesis is just a description of optimal behavior: it does not prescribe how Bayes optimal perception, sensorimotor integration or decision-making under uncertainty emerges. To understand this one has to look at the functional architecture of the brain. Perhaps one of the most remarkable aspects of this architecture is its connectivity. But why does the brain have (axonal and synaptic) connections? Many other functionally specialized organs like the liver or blood do not have a delicate connectivity, so why does the brain? The answer, from point of view of the Bayesian brain, is straightforward: if the brain is making inferences about the causes of its sensations then it must have a model of the causal relationships (connections) among (hidden) states of the world that cause sensory input. It follows that neuronal connections encode (model) causal connections that conspire to produce sensory information. This is an important point, because it means that to understand the Bayesian brain one needs to understand connectivity and the distributed processing that it supports. In short, the Bayesian brain entails an understanding of connectivity and, by implication, functional integration in the brain. This understanding is informed by the fact that functional integration must be optimizing something, in the sense that the Bayesian brain hypothesis just says that perception and decision making are (approximately) Bayes optimal. This is where my story starts, namely, with the inception of functional integration and the attending notion of optimization:

### Prehistory: the functional integration club

In 1990, 2 years into my first research appointment at the MRC Cyclotron Unit in London, I received a phone call from Semir Zeki who asked, “what do you think about integration?” It was an odd question, which I answered politely, from a mathematical perspective. Semir Zeki had just completed a successful collaboration with our fledging (PET) neuroimaging group at the MRC, establishing functional segregation in the human brain using a visual activation paradigm (Lueck et al., 1989). Semir Zeki was (and is) a world-renowned visual neuroscientist, whose physical stature and childlike delight in conceptual challenges are at odds with his intellectual largesse and wisdom (for many, Semir Zeki is the father of modern functional segregation in the brain). Semir has a provocative mischief about him that is impossible to resist. In this instance, Semir wanted to invite Richard Frackowiak and me to a ‘functional integration club’ he had conceived with Horace Barlow. Horace Barlow was an established and respected theoretician, probably most famous for his principle of maximum efficiency (or minimum redundancy) that casts perception in terms of information theory (Barlow, 1961). This club was a remarkable experience for Richard and me (in our late 30s and 20s respectively) and my first exposure to deep conversations about how the brain worked. We met sporadically, in UCL common rooms populated with Formica-topped tables and discarded blackboards or (my favorite) rooms in Cambridge with an old perfume of leather armchair and pipe smoke. The conversation was informal (although I hardly spoke because I was intimidated by Horace Barlow's authority and the fact that he only seemed to smile with his eyes) and wide ranging as was the group's membership. It included people like Graeme Mitchison and a protégé of Horace Barlow's, Peter Földiák. Graeme Mitchison was among the first people to propose an optimality criterion for cortical wiring lengths in the cortex (Durbin and Mitchison, 1990), a theme that resurfaces every few years and has become more acute recently with graph theoretical analyses of connectivity data. I remember Graeme showing us his simulations with a gentle and repressed excitement in a tiny office that was dwarfed by a (moderate sized and probably arcane) computer. Peter Földiák went on to become one of the key players in information theoretic formulations of visual processing and the importance of lateral brain connections in forming orthogonal (sparse) representations (Földiák, 1990). There were many themes discussed in these meetings that have stayed with me for decades. From the point of view of this story, the two key themes were the distinction between functional segregation and functional integration and the notion of optimality. I do not know how or why Semir and Horace chose that group (or that time) to have these seemingly undirected discussions but, from my point of view, they were visionary. First, the dialectic between the brain's attempt to segregate and integrate neatly accommodated our empirical efforts to analyze neuroimaging data in terms of regionally specific activations and their interactions mediated by effective connectivity (the former becoming Statistical Parametric Mapping and the latter Dynamic Causal Modeling). The notion of optimization was, theoretically, central here; whether it was the optimization of cortical wiring lengths or mutual information between sensory input and neuronal responses, the underlying message was that the brain was optimal in some sense. But what was being optimized? Whatever the answer, it was clear from Horace Barlow's work that it should be quantified in terms of information theory or, more simply, probability measures. At this stage, Bayes optimality was not an accepted currency in these conversations and it would be two decades before the equivalence between principle of minimum redundancy and Bayesian treatments of sensory processing would become formally apparent (at least to me; Friston, 2010). When one thinks about optimality in decision making and motor control, one usually turns to optimal control theory. This brings us to next part of my story and the other side of the Bayesian brain, namely, optimal decision theory and value learning. However, my introduction to this was not straightforward.

### History: optimality, natural selection and value

Twenty years ago, I was seconded to the Neurosciences Institute under the Directorship of Gerry Edelman. The Neurosciences Institute is associated with the Neuroscience Research Program (founded in 1962, a few years before the Society for Neuroscience in 1969). Gerry Edelman is an enigmatic Nobel laureate probably most famous in theoretical neuroscience for Neural Darwinism (Edelman, 1993). Marc Raichle once described Gerry Edelman as a “complicated man.” Marc said this with a smile that belied the kindness of his description. In fact, Edelman was (is) a brilliant man with an incisive (invasive) manner that is almost malignant. The atmosphere the Neurosciences Institute was a world away from the armchairs of Cambridge. It was exhilarating and oppressive, echoing its Manhattan environs (before the days of zero tolerance). Edelman himself had an incredible presence and imbued our working atmosphere with an almost homophilic intensity. He had a remarkable and unique perspective on the natural world, which he articulated with nuance and craft. I recall daily lunches in New York cafes, enthralled by his insights and stories. I heard many years later, from Read Montague, that even his jokes were crafted—Read found him memorizing “One Hundred and One Jewish Jokes” as they were disembarking an internal flight. Whether this is true or not, I do not know but it speaks to the fact that impresarios like Edelman (and many others in this story) are never quite what they seem.

It is remarkable that Edelman attracted young people who have become so prominent in our field (and in this story). On arriving, I replaced Read Montague, who had just left under some deliciously unspecified ‘dark cloud.’ Read had been working on theories of value and optimization, in the context of neuronal group selection. This theory rests on the notion that selective pressure could optimize cortical wiring and connectivity to form neuronal groups (or assemblies); in the same way that natural selection operates on phenotypes in evolution. Read went on to publish, with Peter Dayan and others, seminal work linking dopamine firing to value learning (Montague et al., 1995). Interestingly, as I write this, Read is settling into his new office next to mine and Peter Dayan is now Director of the Gatsby Computational Neuroscience Unit next door, which we will come to later. When I arrived, two key young men were Giulio Tononi and Olaf Sporns. Again, it is remarkable that these two scientists have now become world leaders in their own right, Giulio in the context of consciousness and sleep research, while Olaf invented the connectome (Sporns et al., 2005) and is now a key player in our community. I remember clearly Olaf describing his ambition to use empirical connectivity data to understand the complexity of neuronal dynamics on these structures in 1994. Giulio's work on complexity (Tononi et al., 1994) again addressed the fundamental dialectic between functional segregation and integration and how one can be accommodated optimally in the context of the other. I have often wondered whether the focus on this issue reflected in some way the early friendship between Semir Zeki and Gerry Edelman, which, like all true passions, turned into something much darker and enduring.

As a student of probability theory and quantum physics at Cambridge, I had assumed that all formal theories should, ultimately, be cast in mathematical terms. Edelman, on the other hand, considered this as ‘mathematosis’ and about as desirable as halitosis. When I pointed out, at an early group meeting, the formal links between value learning and dynamic programming there was genuine fear and horror in the room about how Edelman would respond. His response was to rusticate me to the library for a period of 6 months. My task was to sanitize my thinking and immerse myself in the writings of the great biological thinkers from Charles Darwin to Ernst Mayr. I complied with this formative (if somewhat brutal) therapy. Six months later, Edelman presented me with a copy of Mayr's ‘The Growth of Biological Thought’ (1982), which I still treasure today. We then wrote on the neurobiology of value learning (with all the maths in an Appendix; Friston et al., 1994). In one sense, Edelman was right; the deep questions about optimality were embedded in selectionist thinking, population dynamics and self organization. However, his puritanical convictions left others to meet the challenge of relating value to more formal treatments in information and probability theory. An approach one could now understand as the Bayesian brain. This brings us to the mid 90s and the rise of Bayesian thinking:

### The Bayesian paradigm

We now move on to 1994, which found me back in London at the opening of the Functional Imaging Laboratory at Queen square (that Richard Frackowiak had moved from the MRC Unit at the Hammersmith Hospital). This was an exciting time: Tim Shallice had overseen the inception of the Institute of Cognitive Neuroscience, which co-occupied a neighboring building with the Gatsby Computational Neuroscience Unit, directed by Geoffrey Hinton. Like Semir Zeki, Geoffrey Hinton exudes a sense of childlike enthusiasm for new insights and conceptual toys. His lectures were considered and profoundly engaging but could not disguise a breathless impatience to get to the point. For me, and I suspect many others, Geoffrey Hinton's ideas placed the Bayesian brain center stage in a tangible and formal fashion. In more general terms, Bayesian formulations of problems in machine learning provided an inescapable metaphor for neuronal computations (e.g., Hinton and van Camp 1993). Notions like the Helmholtz machine and the central role of generative models not only became a natural way of thinking about the brain but also prescribed a principled approach to data analysis, particularly in the context of the ill posed problems we were dealing with at that time. Geoffrey Hinton presented himself with an infectious exuberance but there was also a touch of pathos about him. One story, which I cannot forget, is that he became increasingly unhappy living in London: he had chosen to live in a culturally lively part of town, which the inhabitants of his building chose to celebrate with loud parties that were not sympathetic to the needs of a quiet academic. Hinton's solution to this was to build himself a room within a room; a soundproof cage, within which he slept. Here was a man who had an inventive approach to life's little problems. This inventiveness is clearly apparent in the long history of his contributions to machine learning and computational neuroscience. He was deeply committed to the Bayesian perspective and a great advocate for generative models. A more subtle but terribly important contribution was to cast the generally intractable problem of Bayesian inference in terms of optimization. The insight here was that the same problems that Richard Feynman (1972) had solved in statistical physics, using path integral formulations and variational calculus could be applied to the problem of Bayesian inference, namely, how to evaluate the evidence for a model. This is where free energy minimization comes in, the sense that minimizing free energy is equivalent to (approximately) maximizing the evidence for a model. Note again the underlying role of optimization, which here finessed a difficult but fundamental problem in Bayesian inference.

I remember the last time I spoke to Geoffrey Hinton before his return to Toronto (leaving Peter Dayan in charge of the Gatsby). I do not remember why I went to his office (I suspect he had forgotten why he asked me). He was clearly very excited and spent an hour trying to explain a new approach to unsupervised learning based upon products of experts (Hinton, 2002). I left with my head spinning and a sense that I should try and reciprocate his intellectual generosity. I wrote to him shortly after, trying to summarize my thoughts on biological minimization of free energy. I never heard from him, probably because of his return to Canada that was somewhat complicated by his refusal to fly in airplanes. The notes I sent him were eventually published as the free energy principle about 4 years later (Friston, 2005).

### Bayesian brain and optimization

So how do the legacies of functional integration, information theory, value learning and free energy minimization constitute a history of the Bayesian brain in imaging? The answer lies in optimization: all four perspectives rest on optimizing a single quantity-evidence. In information theory, this corresponds to maximizing the mutual information between sensory information and internal representations; in value learning and selection, the optimization is in terms of value or adaptive fitness, while free energy minimization optimizes the evidence or marginal likelihood of a model. All these processes are the same thing. In other words, maximizing the evidence for a model maximizes the mutual information between successive samples and internal representations (under complexity constraints). This is exactly consistent with the principles of maximum efficiency or minimum redundancy (complexity). But why would Bayesian model evidence be equivalent to adaptive fitness or value? The answer is simple but abstract and again calls on information theory and statistical physics: it turns out that time average of Bayesian model evidence is the same as the (negative) entropy of sensory data sampled by a brain. This means that a Bayesian brain that tries to maximize its evidence is implicitly trying to minimize its entropy. In other words, it resists the second law of thermodynamics and provides a principled explanation for self organization in the face of a natural tendency to disorder. This means the Bayesian brain gracefully accommodates ensemble or population dynamics in evolutionary thinking within a statistical framework. In functionalist terms, such a self organizing system that minimizes its entropy would appear to be making Bayesian inferences about its sensory exchanges with the environment, which, of course, is just the Bayesian brain hypothesis. So is this the end of the story?

### Epilogue

This is not the end of the story but probably its beginning. It is one thing to understand the fundamental imperative that lies behind the Bayesian brain. It is another to understand how selective pressures at an evolutionary and somatic timescale have shaped its anatomy and physiology to meet this imperative. In short, we come back to the nature of functional integration. There are many compelling schemes that may implement the Bayesian brain. Perhaps the most popular is predictive coding, in which bottom-up prediction errors (reporting on those parts of sensory information that have yet to be explained) are suppressed by top-down predictions (Mumford, 1992). In this context, prediction error can be regarded as free energy, such that minimizing free energy is effectively the same as minimizing prediction error. There are all sorts of interesting issues that arise from these considerations, such as the use of hierarchical models and their relationship to the functional logic of cortical connections (e.g., Zeki and Shipp 1988). However, I am approaching my word limit for this essay and it is time to close, where it started.

To understand the mechanisms behind the Bayesian brain, one needs to characterize and quantify the underlying message passing and neuronal infrastructures. In other words, the challenge ahead remains one of functional integration and the measurement of effective connectivity. This has been the focus of many researchers over the past decade and, as intimated in the abstract, appeals to exactly the same principles that underlie Bayes optimality per se. This goes beyond dynamic causal modeling and covers any (Bayesian) evidence-based modeling scheme we apply to neuroimaging data. However, the history of functional and effective connectivity in neuroimaging is another story with its own characters, which can be found in the other articles of this special issue.

I appreciate that I have only covered the first 10 (of the 20) years properly and that I have omitted many important people and issues. However, the next part of the story of the Bayesian brain, along with all its architects, is probably best left for the ‘Future of the History of the Bayesian Brain.’
